# Supplementary material for: Ruminal Yeast Strain with Probiotic Potential: Isolation and Characterization and Its Effect on Rumen Fermentation In Vitro
Source: Microorganisms. 2025 May 30;13(6):1270. doi: 10.3390/microorganisms13061270 (PMC12195040; doi:10.3390/microorganisms13061270)
Supplement: Supplementary file 1 [file microorganisms-13-01270-s001.zip › microorganisms-3632009-supplementary.pdf]

Table S1. Composition and nutrient levels of the fermentation substrate (DM basis）

Diet ingredients %		Nutritional Composition	
Alfalfa	15	ME（MJ/kg）	11.48
oats	15	CP（%）	17.31
Corn	49	NDF（%）	24.87
Soybean meal	21	ADF（%）	14.20
		Starch（%）	35.46
Total	100		

Table S2. Tests for yeast screening as probiotic for ruminal fermentation

**Yeast**	Specie	Growth in anaerobic condition	Growth capacity (OD600)	Acid resistance capacity(OD600)		
				pH 5.0	pH 5.4	pH 5.8
NJ-1	*Candida rugosa*	+	1.085	0.652	0.743	0.783
NJ-2	*Candida rugosa*	-	NT	NT	NT	NT
NJ-3	*Pichia kudriavzevii*	+	1.568	1.022	1.062	1.088
NJ-4	*Pichia kudriavzevii*	-	NT	NT	NT	NT
NJ-5	*Candida rugosa*	+	1.671	1.369	1.389	1.532
NJ-6	*Candida rugosa*	+	0.776	NT	NT	NT
NJ-7	*Candida rugosa*	-	NT	NT	NT	NT
NJ-8	*Candida rugosa*	+	1.538	1.256	1.194	1.199
NJ-9	*Candida rugosa*	+	0.852	NT	NT	NT
NJ-10	*Candida pararugosa*	+	1.218	0.564	0.575	0.671
NJ-11	*Trichosporon asahii*	+	1.115	0.741	0.781	0.819
NJ-12	*Candida rugosa*	+	1.355	1.061	1.191	1.154
NJ-13	*Candida tropicalis*	+	1.333	0.688	0.68	0.766
NJ-14	*Pichia kudriavzevii*	+	1.437	0.925	0.952	1.006
NJ-15	*Trichosporon asahii*	+	0.432	NT	NT	NT
NJ-16	*Trichosporon asahii*	-	NT	NT	NT	NT
NJ-17	*Trichosporon asahii*	-	NT	NT	NT	NT
NJ-18	*Candida rugosa*	-	NT	NT	NT	NT
NJ-19	*Magnusiomyces capitatus*	-	NT	NT	NT	NT
NJ-20	*Magnusiomyces capitatus*	-	NT	NT	NT	NT
NJ-21	*Pichia kudriavzevii*	+	0.885	NT	NT	NT

NJ-22	*Trichosporon asahii*	+	1.213	0.535	0.528	0.539
NJ-23	*Yarrowia lipolytica*	+	0.542	NT	NT	NT
NJ-24	*Candida tropicalis*	+	1.252	0.998	1.120	1.127
NJ-25	*Magnusiomyces capitatus*	-	NT	NT	NT	NT
NJ-26	*Candida pararugosa*	+	0.775	NT	NT	NT
NJ-27	*Candida pararugosa*	-	NT	NT	NT	NT
NJ-28	*Candida rugosa*	+	0.638	NT	NT	NT
NJ-29	*Candida rugosa*	+	0.793	NT	NT	NT
NJ-30	*Candida rugosa*	+	0.543	NT	NT	NT
NJ-31	*Candida rugosa*	+	1.225	0.766	0.778	0.810
NJ-32	*Candida rugosa*	-	NT	NT	NT	NT
NJ-33	*Pichia kudriavzevii*	-	NT	NT	NT	NT
NJ-34	*Pichia kudriavzevii*	-	NT	NT	NT	NT
NJ-35	*Pichia kudriavzevii*	+	0.917	NT	NT	NT
NJ-36	*Candida rugosa*	+	1213	0.972	0.952	0.972
NJ-37	*Meyerozyma caribbica*	-	NT	NT	NT	NT
NJ-38	*Trichosporon asahii*	+	0.657	NT	NT	NT
NJ-39	*Meyerozyma caribbica*	-	NT	NT	NT	NT
NJ-40	*Candida tropicalis*	+	0.887	NT	NT	NT
NJ-41	*Pichia kudriavzevii*	+	1.654	1.023	1.164	1.271
NJ-42	*Sporidiobolus pararoseus*	-	NT	NT	NT	NT
NJ-43	*Trichosporon asahii*	-	NT	NT	NT	NT
NJ-44	*Pichia kudriavzevii*	+	0.654	NT	NT	NT
NJ-45	*Candida rugosa*	+	0.735	NT	NT	NT
NJ-46	*Trichosporon asahii*	+	1.205	1.046	0.999	1.050
NJ-47	*Candida tropicalis*	-	NT	NT	NT	NT
NJ-48	*Meyerozyma caribbica*	-	NT	NT	NT	NT
NJ-49	*Candida tropicalis*	+	0.695	NT	NT	NT
NJ-50	*Pichia kudriavzevii*	-	NT	NT	NT	NT
NJ-51	*Candida tropicalis*	-	NT	NT	NT	NT
NJ-52	*Trichosporon asahii*	+	0.762	NT	NT	NT
NJ-53	*Magnusiomyces capitatus*	-	NT	NT	NT	NT
NJ-54	*Candida pararugosa*	-	NT	NT	NT	NT
NJ-55	*Candida rugosa*	+	1.132	0.569	0.682	0.719
NJ-56	*Candida rugosa*	+	1.376	0.962	1.041	1.216
NJ-57	*Candida rugosa*	-	NT	NT	NT	NT
NJ-58	*Pichia kudriavzevii*	+	1.363	0.679	0.703	0.773
NJ-59	*Pichia kudriavzevii*	+	0.732	NT	NT	NT

“+”:Capable of anaerobic growth, “-”:Not capable; NT:Not tested.
